# Unraveling the role of fibroblasts, FGF5 and FGFR2 in HER2-targeted therapies resistance and tumor progression

**DOI:** 10.18632/oncotarget.27829

**Published:** 2020-12-08

**Authors:** Paloma Bragado, Patricia Fernández-Nogueira, Neus Carbó, Pere Gascón

**Keywords:** breast cancer, TAFs, FGFR2, FGF5, trastuzumab

***Comment on***: *Fernández-Nogueira P et al. Tumor-Associated Fibroblasts Promote HER2-Targeted Therapy Resistance through FGFR2 Activation. Clin Cancer Res. 2020; 26:1432–1448. https://doi.org/10.1158/1078-0432.CCR-19-0353. [PubMed]*

The majority of women with HER2-positive breast cancer will initially respond to trastuzumab and/or other HER2-targeted therapies such as pertuzumab, lapatinib, neratinib and trastuzumab emtansine (T-DM1). However, most will ultimately become resistant to first- and later-line therapies, eventually developing metastasis. Unfortunately, in spite of significant research efforts, no predictive biomarkers of resistance have been clinically validated [1]. In fact, despite all our efforts, HER2-positive metastatic breast cancer remains an almost always deadly disease. Therefore, there is an urgent need for identifying new agents able to overcome acquired resistance to HER2-targeted therapies.

The tumor microenvironment is an ecosystem composed of a variety of cell types (e.g., cancer cells, fibroblasts, immune cells, endothelial cells). It is now well established that understanding the complexity of the tumor microenvironment is crucial, since it plays a key role in cancer progression, metastasis, and response to therapies [2]. Stromal fibroblasts are abundant in breast tumors’ microenvironment and are in constant communication with cancer cells, either via direct cell–cell contact or through the secretion of soluble factors able to activate multiple signaling pathways in tumor cells. Actually, soluble factors stemming from tumor associated fibroblasts (TAFs) have been shown to modulate drug resistance [2]. Improving patients’ treatment requires a better understanding of the mechanisms leading to resistance to currently existing anti-HER2 agents and a deeper comprehension of the role played by the microenvironment-tumor crosstalk.

**Figure 1:**
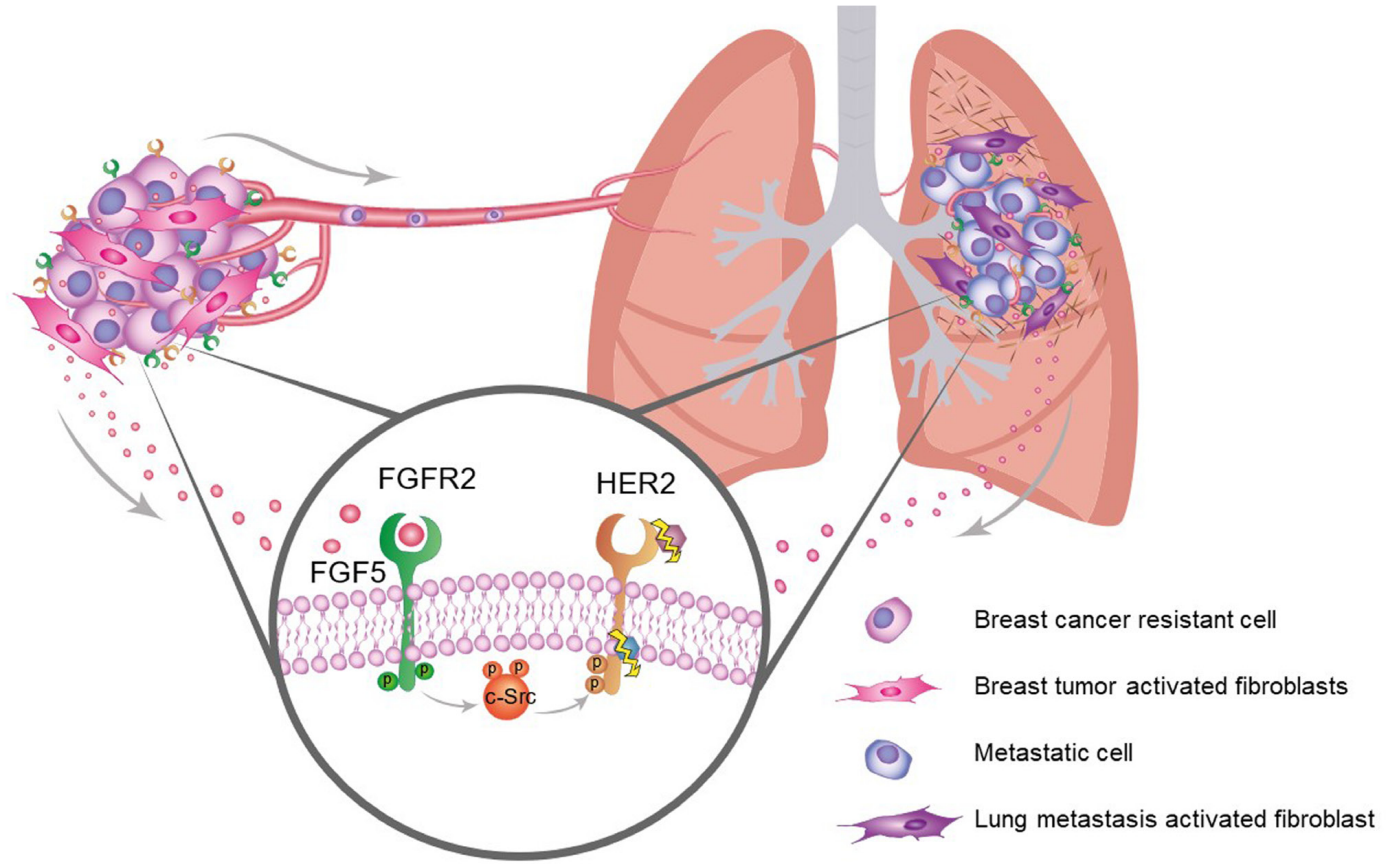
Schematic illustration depicting the crosstalk between tumor activated fibroblasts and breast cancer cells. Breast cancer cells recruit healthy stroma fibroblasts and activate them. Tumor activated fibroblasts then produce FGF5, that will bind to FGFR2 in cancer cells and transactivate HER2 via c-Src. This will promote resistance to HER2 targeted therapies, such as trastuzumab and lapatinib. These resistant cells will disseminate and colonize other organs such as lungs leading to metastasis formation. The FGF5/FGFR2/cSRC/HER2 axis might also promote survival of metastatic cells at secondary organs.

To that end, we have developed several cellular models of resistance to trastuzumab and lapatinib to study the influence of fibroblasts on the response to HER2-targeted therapies. Our aim has been to identify new therapeutic targets and biomarkers of acquired resistance. In our recent paper published in Clinical Cancer Research we have described that TAFs promote HER2-targeted therapy resistance through FGFR2 activation in breast cancer cells [3] (Figure 1). This activation is mediated by the production of soluble FGF5 by the neighboring fibroblasts (Figure 1). We have also described that breast cancer resistance is fueled by HER2 oncogenic addiction. HER2 will remain active in the resistant cells through FGF5/FGFR2 transactivation via c-SRC [3] (Figure 1). Moreover, we also found that resistant cells secrete factors that promote fibroblasts activation generating a positive feedback loop that drives resistance. Furthermore, the coevolution of sensitive breast cancer cells and TAFS *in vivo* also recapitulates the unveiled crosstalk, promoting the selection of a more aggressive and resistant populations, which might also be responsible for future recurrences at secondary organs [3] (Figure 1).

In agreement with our results, several other reports have also proposed that TAFs play an important role in the development of resistance to HER2-targeted therapies in breast cancer [4, 5]. A recent report by Zervantonakis et al. (2020) has described that fibroblast-secreted factors induce survival in response to lapatinib and its combination with either MTOR, BCL-2/XL, or MCL-1 inhibitors restores lapatinib drug sensitivity [6]. Interestingly, in our study the use of FGFR2 inhibitors induced apoptosis in the resistant population inhibiting HER2 phosphorylation, and therefore resensitizing cells to HER2-targeted therapies both *in vitro* and *in vivo*. Hence, our results point out the combination of FGFR2 inhibitors and HER2-targeted drugs as a rational approach to sensitize fibroblast-protected HER2+ breast tumors to HER2-targeted therapies, by shortcutting the TAF/breast cancer cells interplay. In recent years, other studies have linked FGFRs activation to HER2-targeted therapies’ resistance, not only in breast cancer but also in gastric and esophageal cancer [7–9]. These has whipped up interest in testing the combination of FGFR inhibitors with HER2 targeted therapies in the clinical practice. For instance, FiGhTeR trial is a phase II trial that aims to assess the safety and activity of the FGFR (1, 2 and 3) inhibitor pemigatinib, in HER2-trastuzumab resistant gastric cancer patients [10]. To our knowledge, this is the first clinical trial testing the combination of HER2 targeted therapies and FGFR inhibitors although our preclinical studies, among others, strongly suggest that these drug combinations may overcome HER2-targeted therapies resistance. Hence, we foresee the development of new clinical trials testing FGFR inhibitors in patients resistant to HER2-targeted therapies.

Acquired resistance to therapies represent a major obstacle towards finding an effective cancer treatment, accounting for treatment failure in most cases. Given the lack of accurate biomarkers of response, it is unknown which percentage of these failures is due to acquired or intrinsic resistance to treatment. A deep look at our findings in patients’ cohorts has revealed that, fibroblast composition (α-SMA staining), stromal FGF5 and FGFR2 expression contribute to poor outcome and correlate with c-Src activation in HER2-positive patients [3]. This suggests that the TAF/FGF5/FGFR2/c-Src/ HER2 resistance axis that we propose is present in breast cancer patients and it could potentially be reversed with the use of FGFR inhibitors. In terms of response biomarkers, our data also shows that in patients treated with neoadjuvant trastuzumab, FGF5 expression either alone or together with phospho-HER2 expression correlates with a reduced complete response rate, as 98.8% of patients with complete response show low levels of FGF5 and phospho-HER2 expression [3]. Thus, we have identified a new TAF specific biomarker, FGF5, whose expression could be used to predict HER2-targeted therapies response. In agreement with our results Cazet et al. (2018) has shown that triple negative breast cancer cells production of Hedgehog ligand reprograms TAFs to provide a supportive niche for the acquisition of a chemo-resistant phenotype via FGF5 production [11]. Hence, our studies and others have unveiled a relevant role for FGF5 in the stromal regulation of HER2+ cancer cells resistance. They also underline FGF5 inhibition or blockade as an exciting, new and worth-exploring therapeutic strategy for resistant or metastatic HER2 breast cancer, which should proceed to prospective assessment in clinical trials. Interestingly, the therapeutic efficacy of FGF5 vaccines in kidney cancer is being tested in a currently ongoing phase II clinical trial [12].

Our work provides evidence of the role of TAFs, FGF5 and FGFR2 in the resistance to HER2-targeted therapies (Figure 1). We have identified FGFR2 and FGF5 as potential therapeutic targets in resistant HER2-positive breast cancer and we propose stromal FGF5 expression as a new prognosis biomarker for HER2 positive patients. We are now exploring the role of FGFR2 and FGF5 in metastatic HER2-positive breast cancer (Figure 1). We envision that further efforts towards unraveling the role of TAFs/FGF5/FGFR2/HER2 axis in cancer cell dissemination, colonization, and regulation of stemness will help us blunt or prevent metastasis by improving HER2-positive breast cancer patients’ treatment options and survival rates.
